# Metabolomic and transcriptomic analyses of mutant yellow leaves provide insights into pigment synthesis and metabolism in *Ginkgo biloba*

**DOI:** 10.1186/s12864-020-07259-6

**Published:** 2020-12-02

**Authors:** Yaqiong Wu, Jing Guo, Tongli Wang, Fuliang Cao, Guibin Wang

**Affiliations:** 1 Co-Innovation Center for Sustainable Forestry in Southern China, Nanjing Forestry University, 159 Longpan Road, Nanjing, 210037 China; 2 Department of Forest and Conservation Sciences, Faculty of Forestry, The University of British Columbia, Vancouver, V6T 1Z4 Canada; 3 Research Center for Pomology, Institute of Botany, Jiangsu Province and Chinese Academy of Sciences, Qian Hu Hou Cun No.1, Nanjing, 210014 China

**Keywords:** Photosynthesis, Chlorophyll, Carotenoid, Differentially expressed genes

## Abstract

**Background:**

Ginkgo (*Ginkgo biloba* L.) is an excellent landscape species. Its yellow-green leaf mutants are ideal materials for research on pigment synthesis, but the regulatory mechanism of leaf coloration in these ginkgo mutants remains unclear.

**Results:**

We compared the metabolomes and transcriptomes of green and mutant yellow leaves of ginkgo over the same period in this study. The results showed that the chlorophyll content of normal green leaves was significantly higher than that of mutant yellow leaves of ginkgo. We obtained 931.52M clean reads from different color leaves of ginkgo. A total of 283 substances in the metabolic profiles were finally detected, including 50 significantly differentially expressed metabolites (DEMs). We identified these DEMs and 1361 differentially expressed genes (DEGs), with 37, 4, 3 and 13 DEGs involved in the photosynthesis, chlorophyll, carotenoid, and flavonoid biosynthesis pathways, respectively. Moreover, integrative analysis of the metabolomes and transcriptomes revealed that the flavonoid pathway contained the upregulated DEM (−)-epicatechin. Fourteen DEGs from the photosynthesis pathway were positively or negatively correlated with the DEMs.

**Conclusions:**

Our findings suggest a complex metabolic network in mutant yellow leaves. This study will provide a basis for studies of leaf color variation and regulation.

**Supplementary Information:**

The online version contains supplementary material available at 10.1186/s12864-020-07259-6.

## Background

Ginkgo (*Ginkgo biloba* L.) is a one of the oldest dioecious gymnosperms [1], with a history dating back to approximately 200 million years ago [2, 3]. It is an important multipurpose tree species with medicinal and ornamental value [4]. The medicinal value of ginkgo is mainly due to the secondary metabolites of this plant, such as the flavonoids and terpene lactones in its leaves [5]. Research has shown that the secondary metabolites or active ingredients of ginkgo can enhance memory and have favorable therapeutic effects on neurodegenerative diseases [6]. Moreover, ginkgo trees have a beautiful crown, a unique fan-like leaf shape and brilliant leaf color and are susceptible to only a few pests and diseases. Ginkgo is an excellent landscape species, especially for its golden leaves in autumn, and has been widely planted all over the world [7]. A pigment-deficient mutant of ginkgo, which exhibited a yellow-green leaf phenotype on the main branch, was discovered and initially identified as a xantha mutant [8].

Leaf color is not only a phenotypic characteristic that can be easily identified but also an important characteristic of ornamental plants. Leaf color mutants are ideal materials for studying chloroplast development, photosynthesis and light morphogenesis in plants [9, 10]. These mutants may spontaneously occur in nature, be artificially induced or form by the overexpression or silencing of a single or multiple genes via biological engineering [11, 12]. Increasing attention has been directed toward the utilization of leaf color mutants. In tree breeding, leaf color variation can be used as a marker to simplify the breeding of improved varieties and the production of hybrid varieties [13]. Some leaf color mutants have special characteristics, providing high-quality germplasm resources for genetic breeding [14]. These mutants can also be used to analyze and identify gene functions [15] and interactions [16] and to cultivate ornamental plants [17].

Chlorophyll, carotenoids and anthocyanins are the main constituent molecules in the leaves of plants. Leaf yellowing is generally believed to be caused by the degradation of chlorophyll pigments because chlorophyll is the dominant pigment in normal green leaves [18]. Therefore, studies of leaf yellowing mainly focus on chlorophyll biosynthesis (photosynthesis) and degradation. In recent years, many yellow-green leaf color mutants have been found in plants, including *Arabidopsis thaliana* [19], *Zea mays* [20] and *Oryza sativa* [21]. Previous studies have provided an understanding of the proteomic, cytological, physiological and transcriptomic changes in the leaves of the xantha mutant of ginkgo [8, 18]. However, due to the lack of metabolomic analyses, the mechanism underlying leaf coloration in ginkgo remains poorly understood.

In this study, we first compared the chlorophyll content among differently colored leaves of ginkgo. We systematically identified and investigated differences in gene expression and metabolism and screened differentially expressed genes (DEGs) and differentially expressed metabolites (DEMs) associated with pigment biosynthesis and metabolism. Furthermore, we combined transcriptomic and metabolomic analyses to further understand associations between DEGs and DEMs. We anticipated that our results may reveal the physiological, metabolomic and transcriptomic aspects of xantha mutant leaf coloration in ginkgo and provide a useful reference for improvement of leaf color as an ornamental trait.

## Results

### Physiological and metabolic changes

The color of a plant is an external representation of its pigment composition. To better understand the relationship between the color and pigment content of ginkgo leaves, the chlorophyll contents of mutant yellow leaves and normal green ginkgo leaves were measured in this study. The chlorophyll content differed significantly between mutant yellow leaves and normal green leaves of ginkgo. The levels of chlorophyll a and chlorophyll b in the green leaves of ginkgo were 1.34 mg g^− 1^ and 0.75 mg g^− 1^, respectively. In contrast, the levels of chlorophyll a and chlorophyll b in the variant yellow leaves were significantly lower (by 13.43 and 13.33%, respectively) (Table 1).

**Table 1:** Physiological indexes of green and yellow ginkgo leaves

Component	Green leaves	Yellow leaves
Chlorophyll a (mg·g^− 1^)	1.34 ± 0.08^**^	1.16 ± 0.07
Chlorophyll b (mg·g^− 1^)	0.75 ± 0.06^**^	0.65 ± 0.05
Chlorophyll a + b (mg·g^−1^)	2.51 ± 0.14^**^	1.39 ± 0.11

All values are presented as the mean values of three replicates (mean ± SD), and significant differences between green and yellow ginkgo leaves detected using Duncan’s method are indicated by ^**^ (*p* < 0.01).

**Fig. 1:**
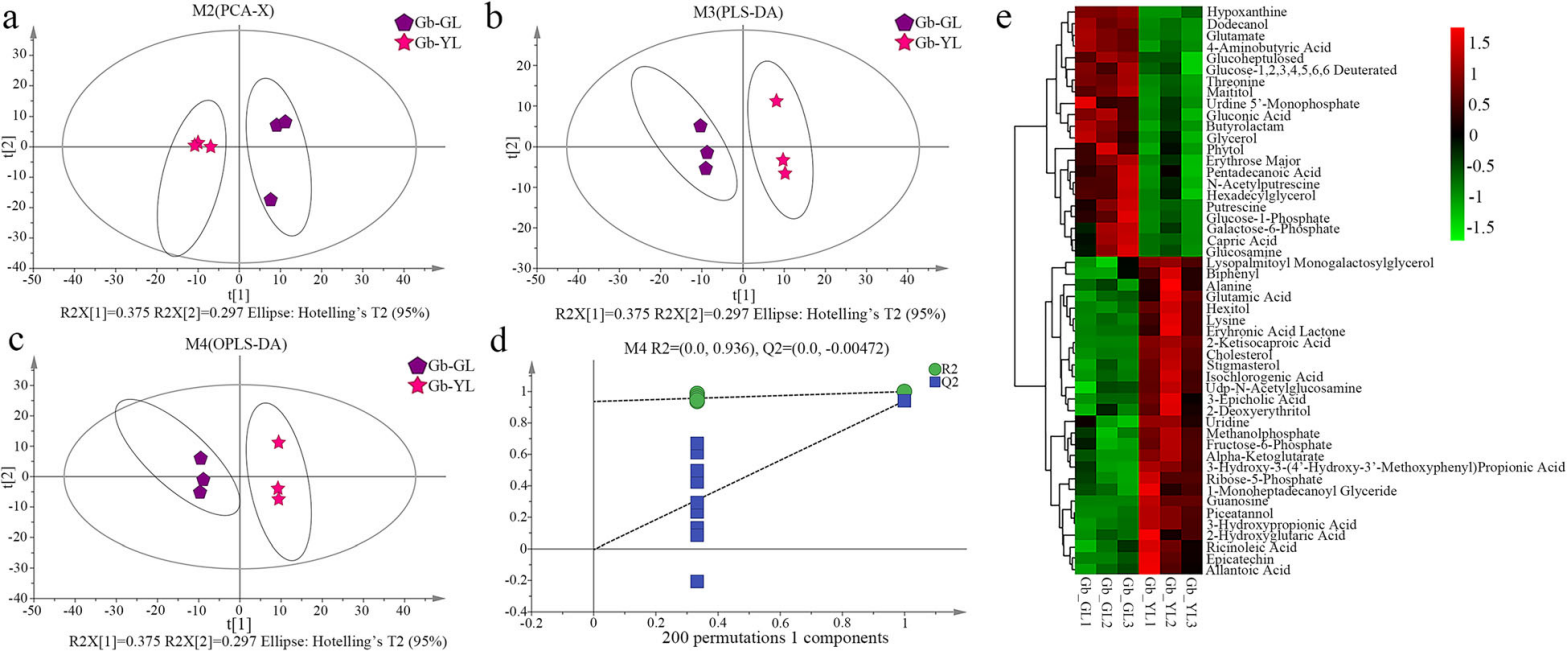
Metabolomic profiling of green and yellow leaves of ginkgo. Principal component analysis (PCA) plots **a**, partial least squares discriminant analysis (PLS-DA) **b**, and orthogonal partial least squares discriminant analysis (OPLS-DA) scores **c** for the analysis of metabolites in green and yellow leaves. **d**. The 200-response sorting tests of the OPLS-DA model. **e**. Fifty differentially expressed metabolites (DEMs) between green and yellow leaves

Metabolomic analysis of different groups (green leaves and yellow leaves) was based on the nontargeted gas chromatography-mass spectrometry (GC-MS) metabolomic method. Analysis of all the samples revealed strong peak capability and retention time reproducibility as well as the total ion current for all the samples (Fig. S1). Data quality control and normalization were performed using an internal standard, and redundancy was eliminated; a total of 283 substances were ultimately detected. The overall distribution among samples and the stability of the entire analytical process were then examined by unsupervised principal component analysis (PCA), followed by supervised orthogonal partial least squares discriminant analysis (OPLS-DA). Fifty DEMs in the metabolic profiles were identified between the two groups (yellow and green leaves) (Fig. 1e). Via PCA, partial least squares discriminant analysis (PLS-DA), OPLS-DA and permutation of the different leaf color groups, score plots a, b, and c were obtained and are shown in Fig. 1. The 200-response sorting chart of the OPLS-DA model is shown in Fig. 1d. The PCA score map (Fig. 1a) shows significant separation between the two groups of samples, reflecting the metabolic differences between the two groups. In addition, the yellow and green leaves of ginkgo also exhibited significant differences (spectral separation) on the PLS-DA and OPLS-DA score maps (Fig. 1b and c). We ultimately mapped 50 DEMs to the Kyoto Encyclopedia of Genes and Genomes (KEGG) database and obtained enrichment results for metabolic pathways. Using the KEGG pathway mapper function, the metabolic pathway enrichment of the DEMs was based on their up- or downregulation. A total of 29 metabolites were annotated to 46 KEGG pathways, and the metabolic pathways were annotated to 21 metabolites.

### Sequencing and assembly

To obtain an overview of the transcriptomes of the differently colored ginkgo leaves (green and yellow leaves), a large amount of paired-end sample sequencing data were obtained using the Illumina platform. The raw sequencing data were submitted to the Short Reads Archive database under accession number SRP182122. Given the impact of the data error rate on the results, Trimmomatic [22] software was used to perform quality pretreatment of the original data, and the number of reads in the whole quality control process was statistically summarized to obtain 931.52 M clean reads from 6 ginkgo samples. Then, HISAT v2.0.4 software was used to compare the sequences of clean reads with the currently published ginkgo genome to obtain position information compared to the reference genome or gene, as well as information regarding the specific sequence characteristics of the sequencing sample. More than 93% of the sequences could be located on the reference genome (Table S1). The average Q30 percentage and GC percentage were 90.45 and 42.89%, respectively. In addition, we used reference transcripts as libraries and used sequence similarity alignment to calculate the expression abundance of each transcript in each sample. The mRNA expression level box-whisker plot and regional distribution map of the 6 samples are shown in Fig. S2.

### qRT-PCR validation

To verify the unigenes obtained by sequencing and further analyze the reliability of the RNA-seq data in the present study, 10 unigenes were random selected for qRT-PCR detection. The expression profiles of these unigenes are shown in Fig. S3. Overall, the results showed that all the selected genes exhibited similar expression patterns as those observed in the RNA-seq data, despite some differences in expression levels. Correlation analysis indicated significant similarity (R^2^ = 0.896, *p*-value< 0.001) between the qRT-PCR values and RNA-seq data. Therefore, our results provide reliable transcriptomic and expression profile data for further research on key genes in ginkgo.

### DEG identification and GO and KEGG enrichment analyses

**Fig. 2:**
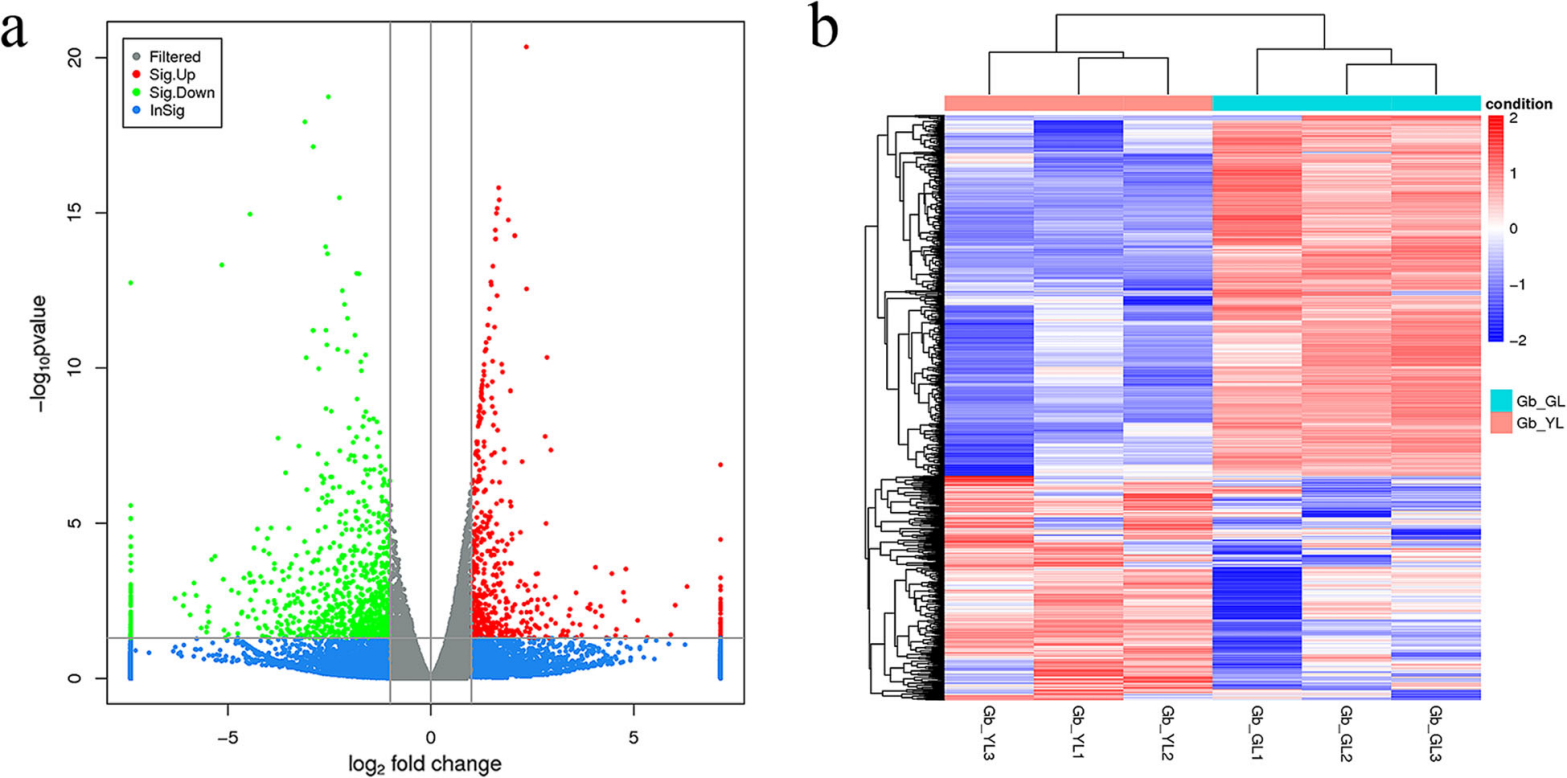
Differentially expressed genes (DEGs) between green and yellow leaves of ginkgo. **a**. DEGs depicted as a volcano plot. The x-axis represents log_2_(fold changes), and the y-axis represents log10 *p*-values. The red points indicate significantly upregulated genes, and the green points represent significantly downregulated genes. **b**. Hierarchical cluster analysis of DEGs. Red indicates significantly upregulated genes, and blue indicates significantly downregulated genes

The fragments per kilobase of transcript per million fragments (FPKM) calculation method was used to analyze gene expression patterns. To identify DEGs between differently colored leaves of ginkgo, we compared the transcript levels of each unigene between green and yellow leaves. A total of 1361 genes were identified, 522 of which genes were significantly upregulated, while 839 genes were significantly downregulated (Fig. 2), indicating that genes showing changes in expression among differently colored leaves were important. The overall mRNA distribution was depicted as a volcano map (Fig. 2a). In the volcano map, gray and blue indicate no significant differences, whereas red and green indicate significant differences in DEGs. In addition, the hierarchical clustering of DEGs showed that samples of the same type cluster together (Fig. 2b), indicating that the genes in the same cluster may have similar biological functions.

**Fig. 3:**
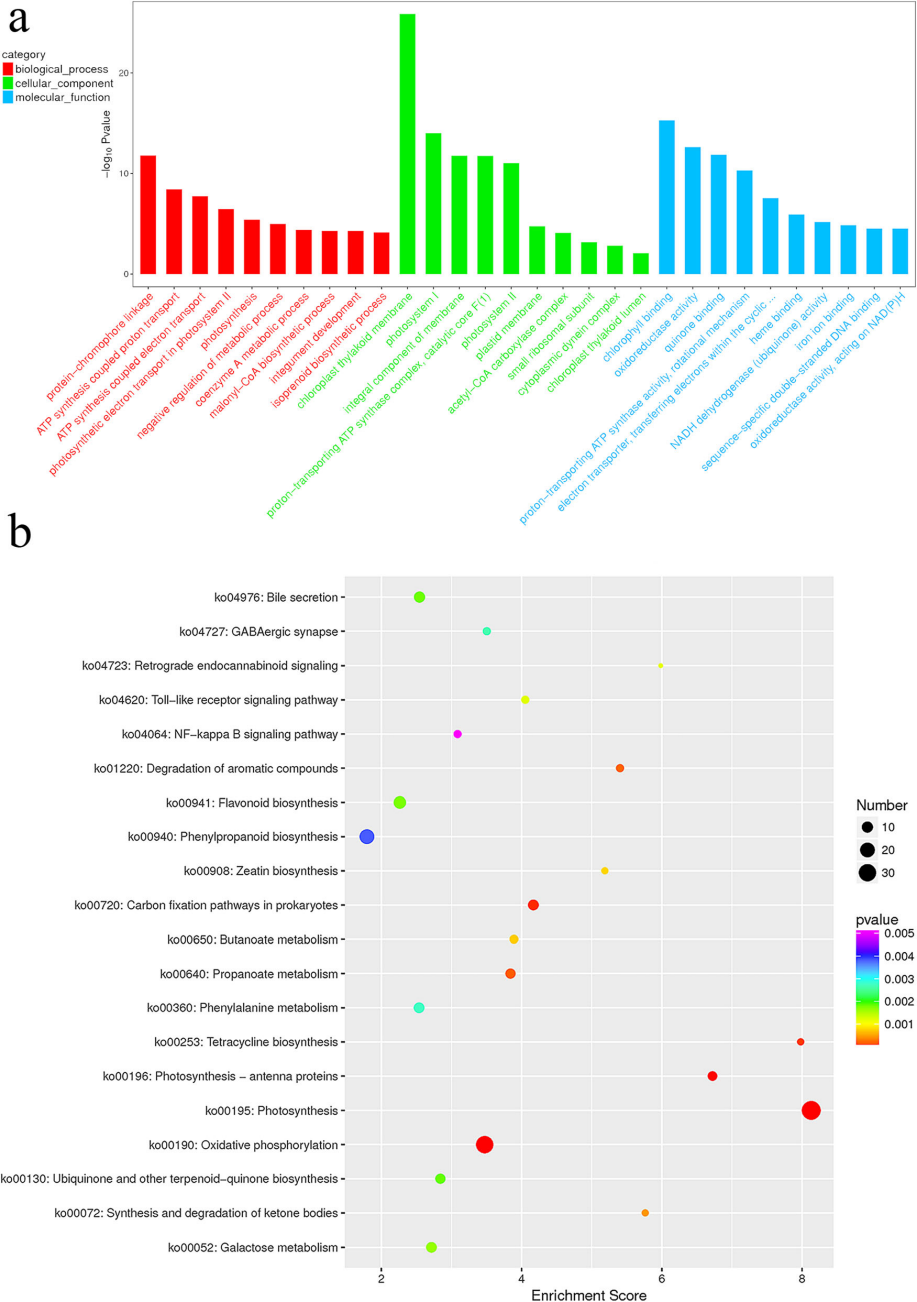
Analyses of the differentially expressed genes (DEGs). **a**. Gene ontology (GO) analyses of the DEGs. The top 30 enriched GO terms. The x-axis shows the names of the GO terms, and the y-axis presents the -log10 p-values. **b**. Kyoto Encyclopedia of Genes and Genomes (KEGG) pathway enrichment of DEGs in ginkgo. The x-axis shows the enrichment score. Entries with larger bubbles contain more DEGs. *P*-values decrease (significance increases) as bubble color changes from purple to blue, blue to green, and green to red

Gene ontology (GO) enrichment analysis was performed on the identified DEGs to elucidate functional differences between differently colored samples. The results showed that the genes involved in “chloroplast thylakoid membrane” and “chlorophyll binding” had the highest degree of enrichment; both of these classifications belong to the cellular component (CC) category and molecular function (MF) category (Fig. 3a). In the biological process (BP) category, “protein-chromophore linkage” exhibited the highest enrichment (Fig. 3a). The Fisher algorithm was used to perform enrichment analyses of BPs, CCs, and MFs for the DEGs of ginkgo, and the GO terms were enriched in the directed acyclic graph of CC. On the basis of KEGG pathway enrichment of the DEGs, the most significantly enriched pathway was photosynthesis (ko00195), followed by oxidative phosphorylation (ko00190) (Fig. 3b).

### DEGs involved in photosynthesis and chlorophyll-, carotenoid- and flavonoid-associated pathways

**Fig. 4:**
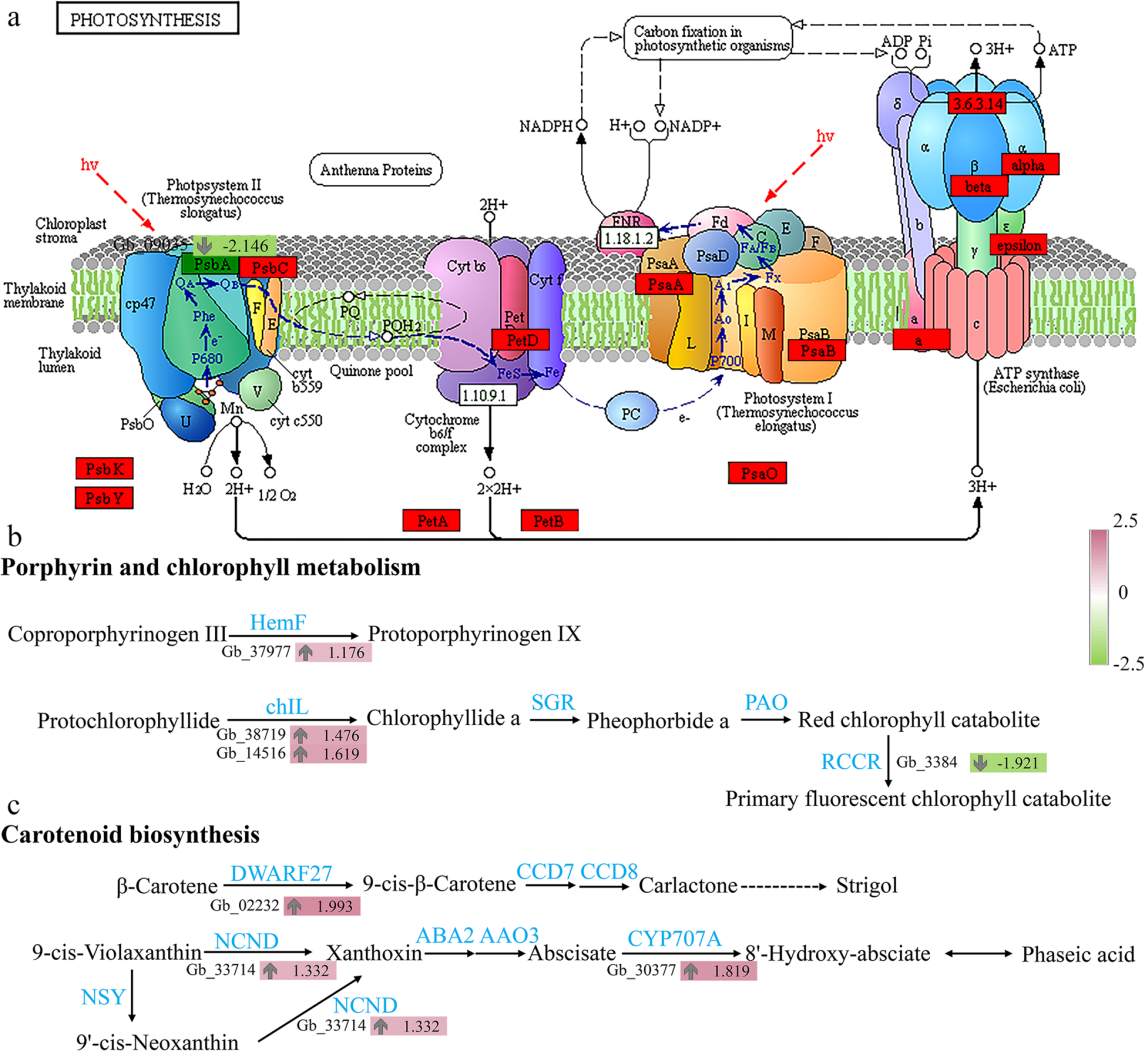
Differentially expressed genes in the photosynthesis, chlorophyll metabolism, and carotenoid biosynthesis pathways. Red indicates upregulated genes, and green indicates downregulated genes. The color is based on log_2_(fold change) values

To identify the structural genes involved in photosynthesis, all the unigenes were searched against the photosynthesis pathway (ko00195) in the KEGG database. A metabolic map was eventually constructed in which multiple transcripts encoding enzymes of the photosynthesis pathway were displayed. Thirty-seven unigene enrichments were noted in the photosynthesis pathway, including upregulated expression of 36 genes and downregulated expression of only one gene, psbA (encoding photosystem II P680 reaction center D1 protein; Gb_09035) (Fig. 4a). In general, photosynthesis and many other metabolic processes occur in chloroplasts, rendering chloroplasts the largest metabolically active regions in leaf cells. Chloroplast development and distribution play an important role in leaf color presentation. In our dataset, four DEGs were annotated as key genes encoding proteins associated with porphyrin and chlorophyll biosynthesis (ko00860). Three unigenes were significantly upregulated in yellow leaves compared with green leaves. The RCCR/ACD2 gene (red chlorophyll catabolite reductase gene; Gb_33842) was downregulated in yellow leaves (Fig. 4b).

**Fig. 5:**
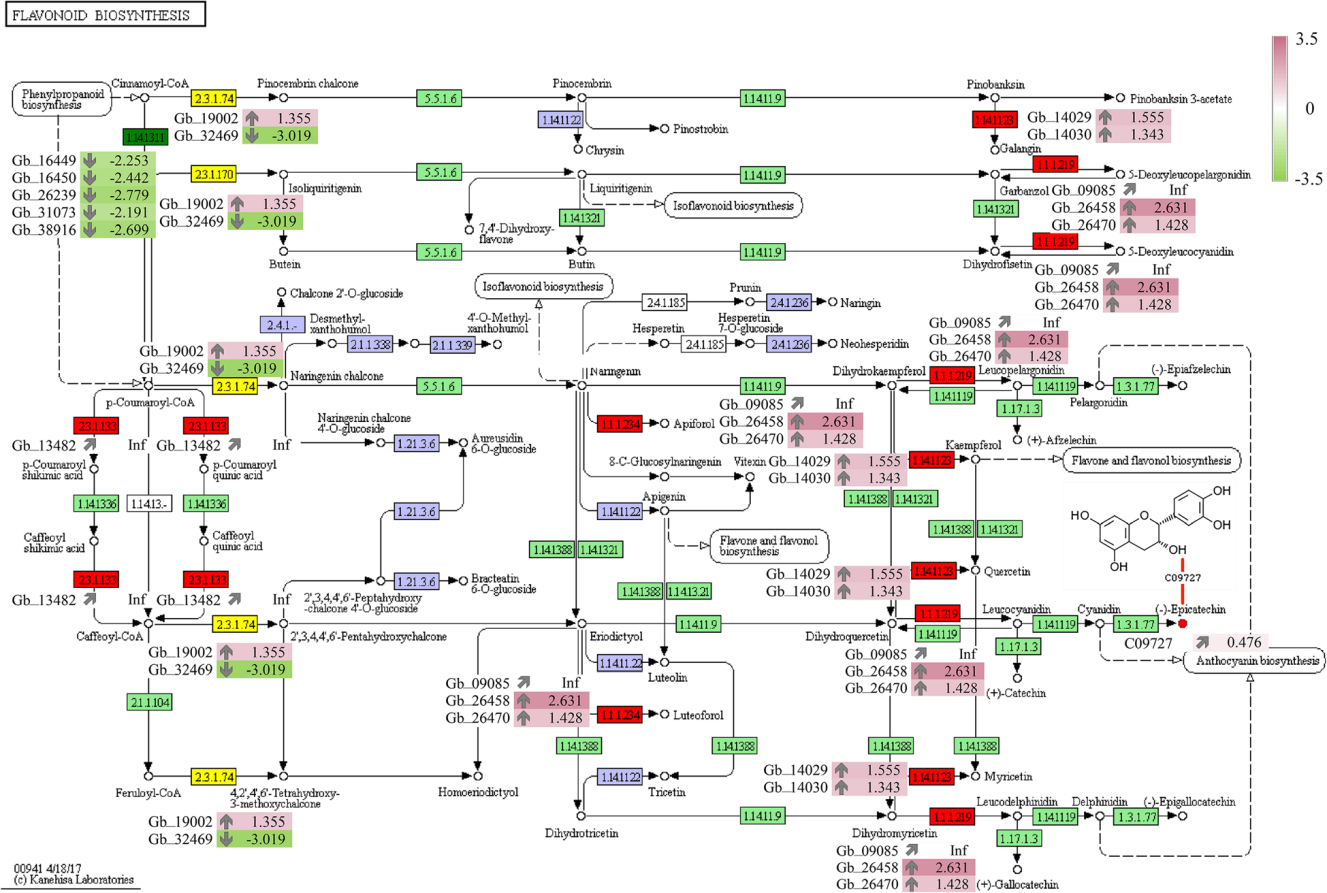
Differentially expressed genes and metabolites in the flavonoid biosynthesis pathway. Boxes represent genes, and circles represent metabolites. Red indicates upregulated genes/metabolites, green indicates downregulated genes/metabolites, yellow indicates both upregulated and downregulated genes/metabolites, and purple-blue indicates that no gene has been annotated. Differentially expressed genes and metabolites are colored based on log_2_(fold change) values

Among the KEGG pathways, we identified three DEGs that were significantly upregulated in association with carotenoid biosynthesis (ko00906) in yellow leaves, including DWARF27 (beta-carotene isomerase; Gb_02232), NCED (9-cis-epoxycarotenoid dioxygenase; Gb_33714), and CYP707A ((+)-abscisic acid 8′-hydroxylase; Gb_09843) (Fig. 4c). Thirty DEGs were annotated as key genes encoding enzymes associated with flavonoid biosynthesis (ko00941). Relative to normal green leaves, yellow leaves exhibited seven DEGs that were significantly upregulated in the flavonoid biosynthesis pathway, namely, CHS (chalcone synthase; Gb_19002), FLS (flavonol synthase; Gb_14029 and Gb_14030), DFR (bifunctional dihydroflavonol 4-reductase/ flavanone 4-reductase; Gb_09085, Gb_26458, and Gb_26470), and HCT (shikimate 0-hydroxycinnamoyltransferase; Gb_13482) (Fig. 5).

### Combined analysis of transcription and metabolism

**Fig. 6:**
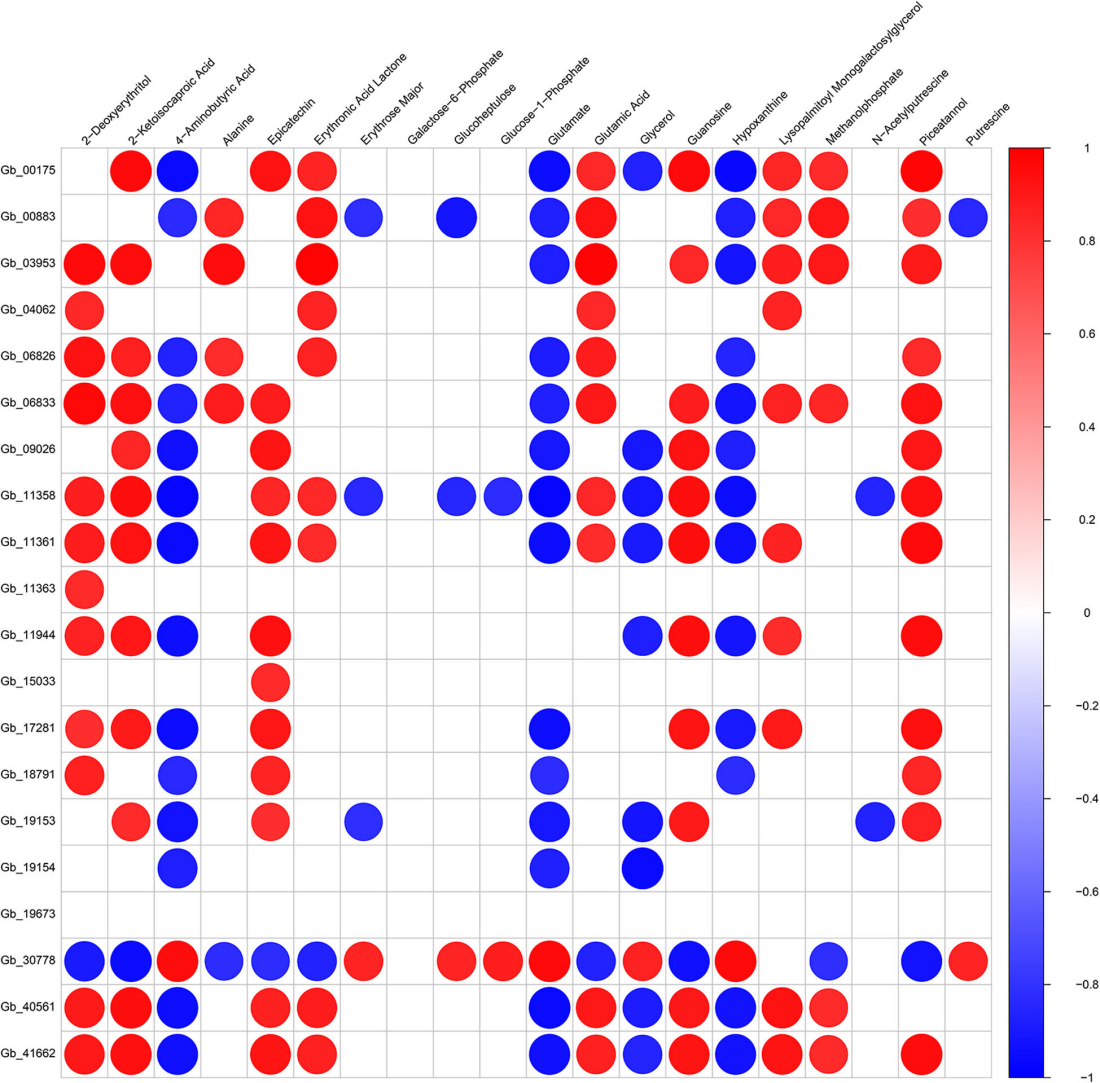
Correlation diagram of differentially expressed genes and metabolites. Each row represents a different gene, and each column is a corresponding metabolite. Red indicates a positive correlation, and blue indicates a negative correlation; a darker color corresponds to a stronger correlation

To further study the differences between green and yellow leaves of ginkgo, the transcriptomic and metabolite data were subjected to correlation analysis. KEGG pathway analysis of the DEGs and DEMs showed that the flavonoid biosynthesis pathway contained the significantly upregulated DEM (−)-epicatechin downstream of ANR (anthocyanidin reductase [EC: 1.3.1.77]; Fig. 5). Moreover, according to the correlation matrix of the top 20 (Fig. 6) results of the correlation analysis of the DEGs and DEMs, a strong correlation was identified between gene expression level and the response strength of the metabolites. Epicatechin had significant positive correlations with 12 DEGs and a negative correlation with only Gb_30778. Fourteen DEGs (Gb_00175, Gb_00883, Gb_03953, Gb_04062, Gb_06826, Gb_06833, Gb_09026, Gb_11358, Gb_11361, Gb_11363, Gb_11944, Gb_18791, Gb_19153, and Gb_41662) from the photosynthesis pathway (ko00195) had positive or negative correlations with the DEMs. Among them, piceatannol had significant positive correlations with 11 photosynthesis biosynthesis DEGs. Erythronic acid lactone had significant positive correlations with 8 photosynthesis biosynthesis DEGs, especially Gb_03953 (correlation = 0.99, *p*-value < 0.001).

## Discussion

### Physiological changes

The formation of yellow-green leaf color mutants is induced by a variety of genetic and environmental factors [8]. In this study, yellow-green leaf color mutants appeared in the same environment, indicating that genetic changes play a decisive role. The formation of leaf color is closely associated with chlorophyll synthesis and transport [23]. Chlorophyll is a key pigment affecting the coloration of plant leaves, and leaf color variation is usually caused by loss of chlorophyll in plants [24, 25]. Chlorophyll, including chlorophyll a and chlorophyll b, mainly exists in the leaves of plants and is distributed in chloroplasts. Chlorophyll isprimarily responsible for the capture and photosynthetic transformation of light energy in plants and is the most important pigment in the processes of light morphogenesis and organic accumulation in plants [26]. Here, by measuring the chlorophyll content, we found that the chlorophyll a or b content in xantha mutant leaves of ginkgo was significantly lower than that in normal green leaves, which affected the normal appearance of leaf color and led to the phenotype of chlorophyll deficiency, i.e., yellow leaves. This finding is similar to the findings of Liu et al. [8] and Li et al. [18]

### DEGs involved in photosynthesis and chlorophyll-associated pathways

RNA-seq and transcriptomic analyses can provide information regarding quantitative changes in gene expression [27, 28]. Therefore, these methods can identify new transcripts and predict the roles of transcripts in leaf coloration. In this study, by performing transcriptome expression profile analysis of normal green leaves and yellow mutant leaves, we identified 1361 DEGs via mapping to the ginkgo reference genome, including 37 unigenes enriched in the photosynthesis pathway. Leaf color mutations can damage chloroplast development, and this type of damage is often considered one of the reasons for changes in photosynthesis [8]. The chloroplast is an important organelle for photosynthesis in plants and is also the site for the synthesis and accumulation of the photosynthetic pigment chlorophyll and carotenoids [29]. We found that four DEGs associated with porphyrin and chlorophyll biosynthesis, including CPOX (coproporphyrinogen III oxidase; Gb_37977) and chlL (light-independent protochlorophyllide reductase subunit L; Gb_38719 and Gb_14516), were upregulated in the yellow leaves. CPOX is a key enzyme in the synthesis of porphyrins, which play an important role in pigment formation [30]. Related studies have shown that porphyrins can be yellow, pink or brown [31, 32]. Hence, the significantly upregulated expression of CPOX may be one of the important reasons for the formation of yellow-leaved mutants. Chlorophyll synthesis in plants is a very complex process, consisting of 16 steps, which are completed by 16 enzymes encoded by more than 20 genes [33]. Mutation of any gene in this pathway may affect the synthesis of chlorophyll, thus changing the content and proportion of various pigments in chloroplasts and ultimately leading to changes in leaf color.

### DEGs associated with carotenoid and flavonoid biosynthesis

In this study, DWARF27 (Gb_02232), a β-carotene isomerase with iron, was significantly upregulated in the yellow leaves. Carotenoids, which can be yellow, orange or red, are among the most important factors underlying differences in color among plant leaves [34, 35]. In addition, flavonoids, a class of polyphenol secondary metabolites, are widely present in various plant organs and are involved in the regulation of color formation in flowers, leaves and fruits [36]. We found that two FLS genes (Gb_14029 and Gb_14030) are significantly upregulated in yellow leaves compared to green leaves and may play an important role in yellow-leaf formation in ginkgo. Several studies have shown that FLS plays important roles in color formation and growth in plants and can protect plants from harm caused by the external environment [37–39].

Plant anthocyanins are a class of water-soluble flavonoid pigments. DFR is the first key enzyme in the downstream pathway of anthocyanin synthesis and is an important regulatory node in the anthocyanin biosynthesis pathway [40, 41]. Here, three DFR genes (Gb_09085, Gb_26458 and Gb_26470) were up-regulated in yellow leaves, which may be conducive to the formation of yellow leaves and the accumulation of anthocyanins in ginkgo.

### Integrative analysis of the metabolome and transcriptome

By GC-MS analysis, we identified 283 metabolites, including 50 significant DEMs. Through integrative analysis of the metabolomes and transcriptomes, we found that the flavonoid biosynthesis pathway contained the significantly upregulated DEM (−)-epicatechin downstream of ANR (Fig. 5). We also found that three DFR genes were significantly upregulated in the yellow leaves. The upregulated expression of these DFR genes might be the main cause of the increase in the downstream (−)-epicatechin content. DFR is also an important regulatory node in the anthocyanin biosynthesis pathway [42]. Therefore, DFR can directly or indirectly affect the synthetic and metabolic pathways of anthocyanins, leading to changes in the (−)-epicatechin content in plants, which ultimately leads to leaf color variation. Integrative analysis of the transcriptome and metabolome can be used not only to study the genetic characteristics of metabolites but also to identify candidate genes associated with metabolism [43].

In addition, chlorophyll, a green pigment that is crucial for photosynthesis, absorbs energy from sunlight in the antenna system and transfers the energy to the reaction center [26, 44]. Fourteen DEGs from the photosynthesis pathway were positively or negatively correlated with the DEMs based on the correlation analysis in this study. We speculated that the reason for this phenomenon may be the spatial and temporal differences in gene expression and metabolism. In general, the accumulation of metabolites in plants is a slow process, and the expression of genes is often instantaneous [45]. Because a large number of genes were differentially expressed during the photosynthetic period, these transiently expressed genes regulate the production of metabolites, resulting in a relative lag in metabolite content.

In summary, leaf color is mainly affected by the contents and distributions of chlorophyll, carotenoids and anthocyanins in cells [46, 47]. Examination of leaf color mutants is an effective method for exploring of gene function during chloroplast development. Therefore, the discovery and identification of leaf color mutant genes and functional studies on chlorophyll-related genes and metabolites are of great significance. This study provides an ideal reference for investigations of the chlorophyll biosynthesis pathway and photosynthetic mechanism of ginkgo. To determine which of the DEGs in yellow leaves can be used as marker genes, we must confirm the main or key genes regulating ginkgo in the future. Moreover, a large number of SNPs are required for correlation analysis with ginkgo yellow/ green leaves and to explore the linkage between SNP loci and key genes. In this study, the DEGs leading to leaf color-related mutations were preliminarily demonstrated, providing a theoretical basis for the production and application of high-quality breeding.

## Conclusions

Yellow-green leaf ginkgo mutants provide ideal materials for studying the regulatory mechanisms of leaf coloration. Through transcriptomic and metabolomic analyses, we identified a total of 1361 DEGs and 283 metabolites (including 50 DEMs), respectively. Notably, 37 of the identified DEGs were enriched in the photosynthesis pathway, 4 in the chlorophyll pathway, 3 in the carotenoid pathway, and 13 in the flavonoid biosynthesis pathway, suggesting that the ginkgo mutant exhibits specific differences in the regulation or alteration of pigment biosynthesis. Integrative analysis of the metabolome and transcriptome showed that the flavonoid pathway contained the significantly upregulated DEM (−)-epicatechin downstream of ANR. Moreover, 14 DEGs from the photosynthesis pathway were positively or negatively correlated with the DEMs. The identification of these differentially accumulated genes and metabolites suggests the presence of a complex regulatory mechanism in the mutant yellow leaves of ginkgo. These results also provide a useful reference for investigations of leaf color variations and the underlying molecular mechanisms in other plants.

## Methods

### Plant material

**Fig. 7:**
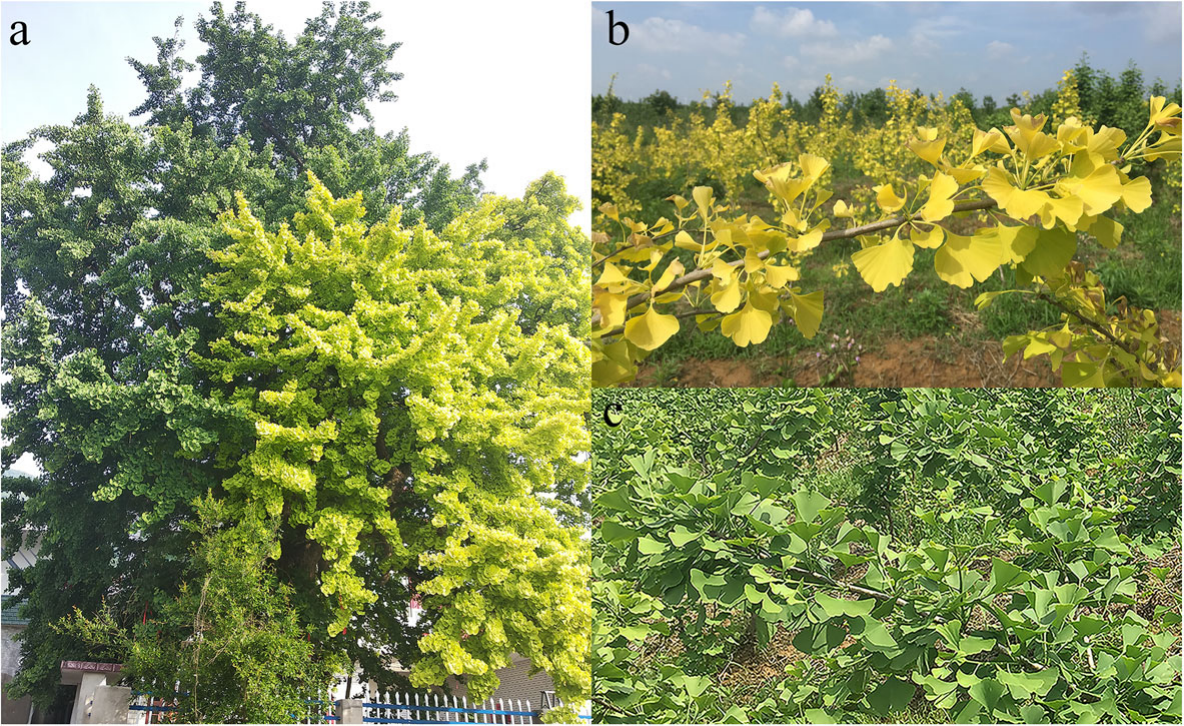
Phenotypes of normal green leaves and mutant yellow leaves of ginkgo. **a**. The xantha mutant is an ancient tree that exhibits a yellow-green leaf phenotype on a main branch. **b**. The phenotype of mutant yellow leaves. **c**. The phenotype of normal green leaves

The pigment-deficient mutant of ginkgo selected as the experimental material was identified in Jiujiang city, Jiangxi Province, China (29°49′ N, 116°40′ E). This xantha mutant is an ancient tree that is estimated to be 150 years old and exhibits a yellow-green leaf phenotype on a main branch [8]. This branch is thought to result from a bud mutation that composes a quarter of the crown, with the remainder of the crown branches producing green leaves (Fig. 7a). We obtained permission to collect ginkgo leaves and branches. Several green leaf and yellow leaf scions were grafted onto rootstocks in the ginkgo germplasm nursery at Nanjing Forestry University Base. Professor Fuliang Cao undertook formal identification of these samples (the xantha mutant is called: *Ginkgo biloba* ‘Wannianjin’) and obtained a Good tree variety certificate (S-SV-GB-008-2014) issued by the State Forestry Administration of China. Ginkgo ‘Wannianjin’ trees are rare, and stable phenotypes are attractive as ornamental resources for cultivation. The phenotypes of mutant yellow leaves and normal green leaves are shown in Fig. 7b and c, respectively. In this study, three leaves were sampled per replicate, with three biological replicates for each group in the same period. Transcriptome sequencing, qRT-PCR validation and metabolite determination were performed in each group using the same plant material, including three biological replicates.

### Physiological and metabolic assays

#### Measurement of physiological indexes

Chlorophyll levels in leaves were measured using corresponding assay kits, namely, a chlorophyll assay kit (A147), according to the manufacturer’s instructions (Jiancheng Bioengineering Inc., Nanjing, China). The experiments were repeated three times. Statistical analyses were performed using SPSS 22.0 software (SPSS Inc., Chicago, IL, USA).

#### Metabolome analyses

A total of 20 mg of accurately weighed ginkgo leaves was subjected to extraction by grinding in 360 μL of cold methanol and ultrasonication for 30 min. Then, 200 μL of chloroform and 400 μL of water were added, and the samples were vortexed. Ultrasonic extraction was performed subsequently for 30 min. The samples were then centrifuged for 10 min at 4 °C and 12,000 rpm. The subsequent steps were performed as described by Ge et al. [48]. The final injection volume was l μL in splitless mode.

Metabolites were annotated using the Fiehn or NIST database. The data were normalized to the total peak area of each sample and multiplied by 10,000, and the peaks from the same metabolite were combined. The data were log_2_- transformed in Excel 2007 (using 0.000001 to replace 0 before transformation). The DEMs were selected on the basis of both a statistically significant threshold of variable influence on projection (VIP) values obtained from an OPLS-DA model and *p*-values from a two-tailed Student’s t-test of the normalized peak areas from different groups; metabolites with VIP values larger than 1.0 and p-values less than 0.05 were considered DEMs [49].

### Transcriptome sequencing

Total RNA extraction and assembly were performed according to the method described by Wu et al. [50] except that sequencing in this study was performed with an Illumina sequencing platform (HiSeq X 10). Clean reads filtered from raw reads were mapped to the ginkgo reference genome (http://gigadb.org/dataset/100209) using HISAT v2.0.4 software.

### Quantitative real-time PCR (qRT-PCR) analysis

The results of the transcriptional analysis were validated by qRT-PCR. First, 1 μg of RNA from each sample was reverse transcribed to first-strand cDNA using Superscript II reverse transcriptase (Invitrogen, Carlsbad, CA, USA) according to the manufacturer’s instructions. Gene-specific primer pairs (Table S2) designed using Oligo 6.0 were used for qRT-PCR. Reactions were performed with SYBR Green Master Mix Rox for RT-PCR Kit (Roche, Indianapolis, IN, USA) on a ViiA 7 System (Applied Biosystems, Foster City, CA, USA). The PCR conditions were as follows: 50 °C for 2 min and 95 °C for 2 min, followed by 45 cycles of 95 °C for 1 s and 60 °C for 30 s. The glyceraldehyde-3-phosphate dehydrogenase gene (amplified with primers F: GGTGCCAAAAAGGTGGTCAT and R: CAACAACGAACATGGGAGCAT) was used as a reference. The relative expression was determined using the 2^−ΔΔCt^ method. Each experiment was performed in triplicate, and the data were subjected to statistical analysis.

## Supplementary Information

**Additional file 1: Table S1.** Primer pairs for quantitative real-time PCR. **Table S2.** Results of the comparison of sequences to the reference genome.

**Additional file 2: Figure S1.** Total ion chromatography (TIC) of metabolites extracted from ginkgo.

**Additional file 3: Figure S2.** mRNA expression level boxplot and regional distribution map. a. Box-whisker plot of FPKM values. Sample names are presented along the abscissa, and log10 (FPKM+ 1) values are presented on the y-coordinate. The box chart for each region corresponds to five statistics (from top to bottom: the maximum, upper quartile, median, lower quartile and minimum). b. FPKM expression profile of each sample. Different colors in the figure represent FPKM values in different ranges. The abscissa presents the sample names, and the ordinate presents the transcript numbers.

**Additional file 4: Figure S3.** qRT-PCR validation of 10 putative genes in the ginkgo transcriptome. a. Histogram showing the qPCR results of 10 unigenes between green leaves and yellow leaves of ginkgo. b. Histogram showing the FPKM values of these unigenes.

## Data Availability

All raw data were submitted to the Short Reads Archive database under the accession number SRP182122.

## Abbreviations

Ginkgo: *Ginkgo biloba* L.
DEGs: Differentially expressed genes
DEMs: Differentially expressed metabolites
GC-MS: Gas chromatography-mass spectrometry
PCA: Principal component analysis
OPLS-DA: Orthogonal partial least squares discriminant analysis
PLS-DA: Partial least squares discriminant analysis
KEGG: Kyoto Encyclopedia of Genes and Genomes
FPKM: Fragments per kilobase of transcript per million fragments
GO: Gene ontology
CC: Cellular component
MF: Molecular function
BP: Biological process
RCCR/ACD2 gene: Red chlorophyll catabolite reductase gene
DWARF27: Beta-carotene isomerase
NCED: 9-cis-epoxycarotenoid dioxygenase
CYP707A: (+)-abscisic acid 8′-hydroxylase
CHS: Chalcone synthase
FLS: Flavonol synthase
DFR: Bifunctional dihydroflavonol 4-reductase/ flavanone 4-reductase
HCT: Shikimate 0-hydroxycinnamoyltransferase
ANR: Anthocyanidin reductase
CPOX: Coproporphyrinogen III oxidase
chlL: Light-independent protochlorophyllide reductase subunit L
VIP: Variable influence on projection
qRT-PCR: Quantitative real-time PCR
