# Supplementary material for: Metabolomic and transcriptomic analyses of mutant yellow leaves provide insights into pigment synthesis and metabolism in Ginkgo biloba
Source: BMC Genomics. 2020 Dec 2;21:858. doi: 10.1186/s12864-020-07259-6 (PMC7709416; doi:10.1186/s12864-020-07259-6)
Supplement: Supplementary file 1 — Additional file 1: Table S1. Primer pairs for quantitative real-time PCR. Table S2. Results of the comparison of sequences to the reference genome. [file 12864_2020_7259_MOESM1_ESM.doc]

**Supplementary information**

**Table S1.** Primer pairs used for quantitative real-time PCR.

| Primer ID | Forward (5’-3’) | Reverse (5’-3’) |
| --- | --- | --- |
| AtHO1 | ATGATAGGGGCAAGGGTAAG | GTGAAGGAAAAGAAGAGCATC |
| FNR | CAGGAGACGAAGCACACGA | TTCCAAAGCGTCAATCTCTCT |
| ODD | CAAACATTAGCAGAGAGTGGA | CCCCAATCTTGTGCTGCTCT |
| FLS1 | TCTTATACCCAATGATGTGCC | TGTCGTTGCTCAATATCTGTAG |
| 1CYP450 | CTGCCTTCCTGTTTTTCTGTT | TCATAATCAGAGTCCGAGGG |
| 2CYP450 | CTTCTCCTCCGTACTTGGTT | ATGGCATCGTGGGTTTTTAGA |
| bHLH82 | AGGGCGTCAGCGGCGTCA | CGTTGTTGTTTGAAGCACTGT |
| bHLH105 | GTTGGAGGAAGTGGGAGTG | CGCATCTTTTCACGGCAAGC |
| DPOR | GTCTTCCATCGTCCGTCTG | GAAAGGATACGAAGTTGAACAT |
| FLS2 | CCGCCCTGAAACCATACCT | GGAGAGATGCCATGATTGAT |

**Table S2.** Results of the comparison of sequences to the reference genome.

| Sample | Gb_GL1 | Gb_GL2 | Gb_GL3 | Gb_YL1 | Gb_YL2 | Gb_YL3 |
| --- | --- | --- | --- | --- | --- | --- |
| Total reads | 155,748,470 | 154,786,762 | 154,842,644 | 156,144,754 | 156,082,008 | 153,919,782 |
| Total mapped reads | 146,108,650  (93.81%) | 145,262,359  (93.85%) | 145,259,173  (93.81%) | 146,231,793  (93.65%) | 146,784,963  (94.04%) | 144,301,055  (93.75%) |
| Multiple mapped reads | 29,529,278  (18.96%) | 31,644,904  (20.44%) | 30,418,178  (19.64%) | 37,605,100  (24.08%) | 36,841,213  (23.60%) | 37,510,106  (24.37%) |
| Uniquely mapped reads | 116,579,372  (74.85%) | 113,617,455  (73.40%) | 114,840,995  (74.17%) | 108,626,693  (69.57%) | 109,943,750  (70.44%) | 106,790,949  (69.38%) |
| Reads mapped to '+' | 57,845,316  (37.14%) | 56,341,044  (36.40%) | 57,057,533  (36.85%) | 53,926,015  (34.54%) | 54,494,179  (34.91%) | 52,935,030  (34.39%) |
| Reads mapped to '-' | 58,734,056  (37.71%) | 57,276,411  (37.00%) | 57,783,462  (37.32%) | 54,700,678  (35.03%) | 55,449,571  (35.53%) | 53,855,919  (34.99%) |
| Nonspliced reads | 95,629,708  (61.40%) | 94,463,633  (61.03%) | 94,620,287  (61.11%) | 93,062,102  (59.60%) | 93,909,901  (60.17%) | 91,822,698  (59.66%) |
| Spliced reads | 20,949,664  (13.45%) | 19,153,822  (12.37%) | 20,220,708  (13.06%) | 15,564,591  (9.97%) | 16,033,849  (10.27%) | 14,968,251  (9.72%) |
| Reads mapped in proper pairs | 103,220,762  (66.27%) | 99,506,186  (64.29%) | 101,185,068  (65.35%) | 92,941,978  (59.52%) | 93,476,014  (59.89%) | 90,869,562  (59.04%) |
