# Supplementary figures and images for: Metabolomic and transcriptomic analyses of mutant yellow leaves provide insights into pigment synthesis and metabolism in Ginkgo biloba
Source: BMC Genomics. 2020 Dec 2;21:858. doi: 10.1186/s12864-020-07259-6 (PMC7709416; doi:10.1186/s12864-020-07259-6)

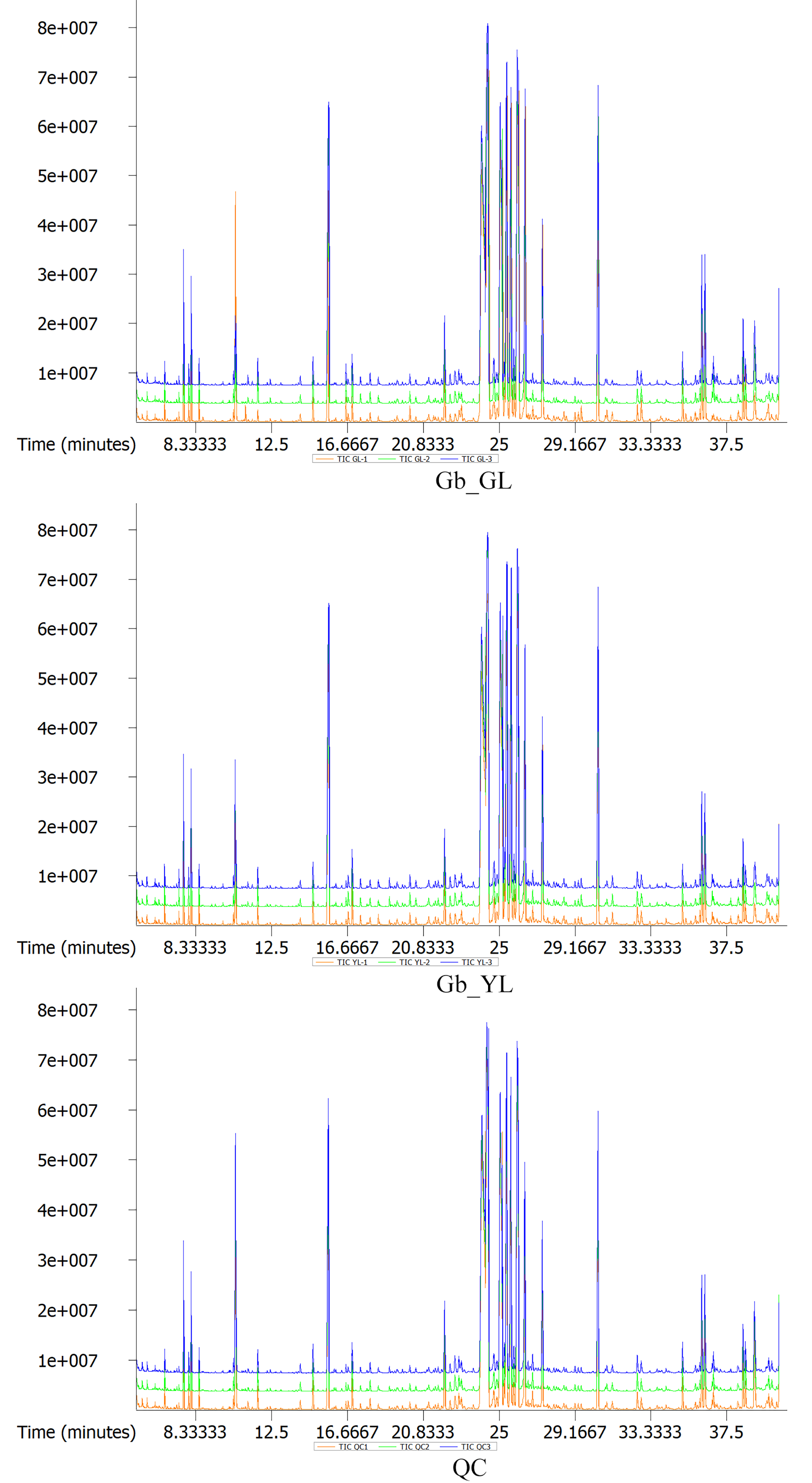

Supplement: Supplementary file 2 — Additional file 2: Figure S1. Total ion chromatography (TIC) of metabolites extracted from ginkgo. [file 12864_2020_7259_MOESM2_ESM.tif]

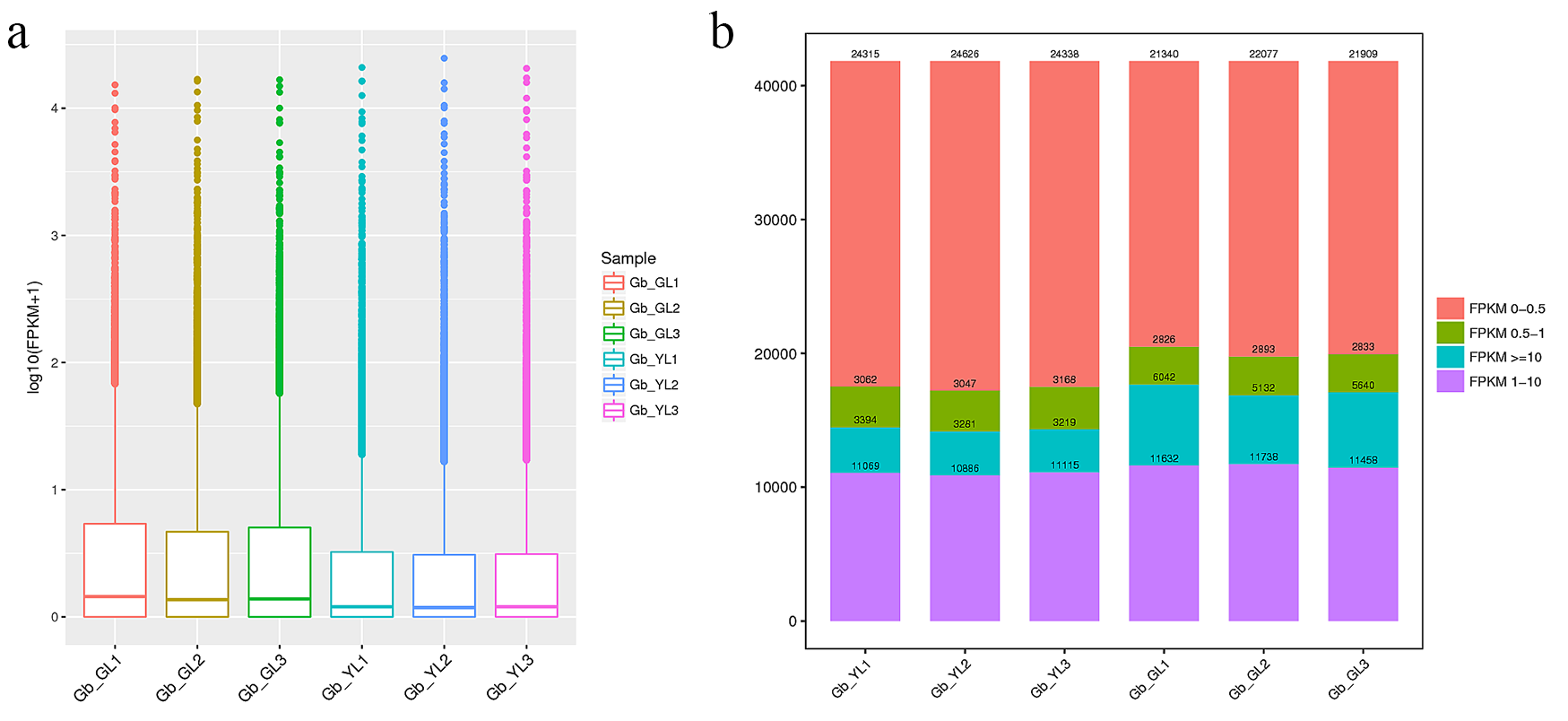

Supplement: Supplementary file 3 — Additional file 3: Figure S2. mRNA expression level boxplot and regional distribution map. a. Box-whisker plot of FPKM values. Sample names are presented along the abscissa, and log10 (FPKM+ 1) values are presented on the y-coordinate. The box chart for each region corresponds to five statistics (from top to bottom: the maximum, upper quartile, median, lower quartile and minimum). b. FPKM expression profile of each sample. Different colors in the figure represent FPKM values in different ranges. The abscissa presents the sample names, and the ordinate presents the transcript numbers. [file 12864_2020_7259_MOESM3_ESM.tif]

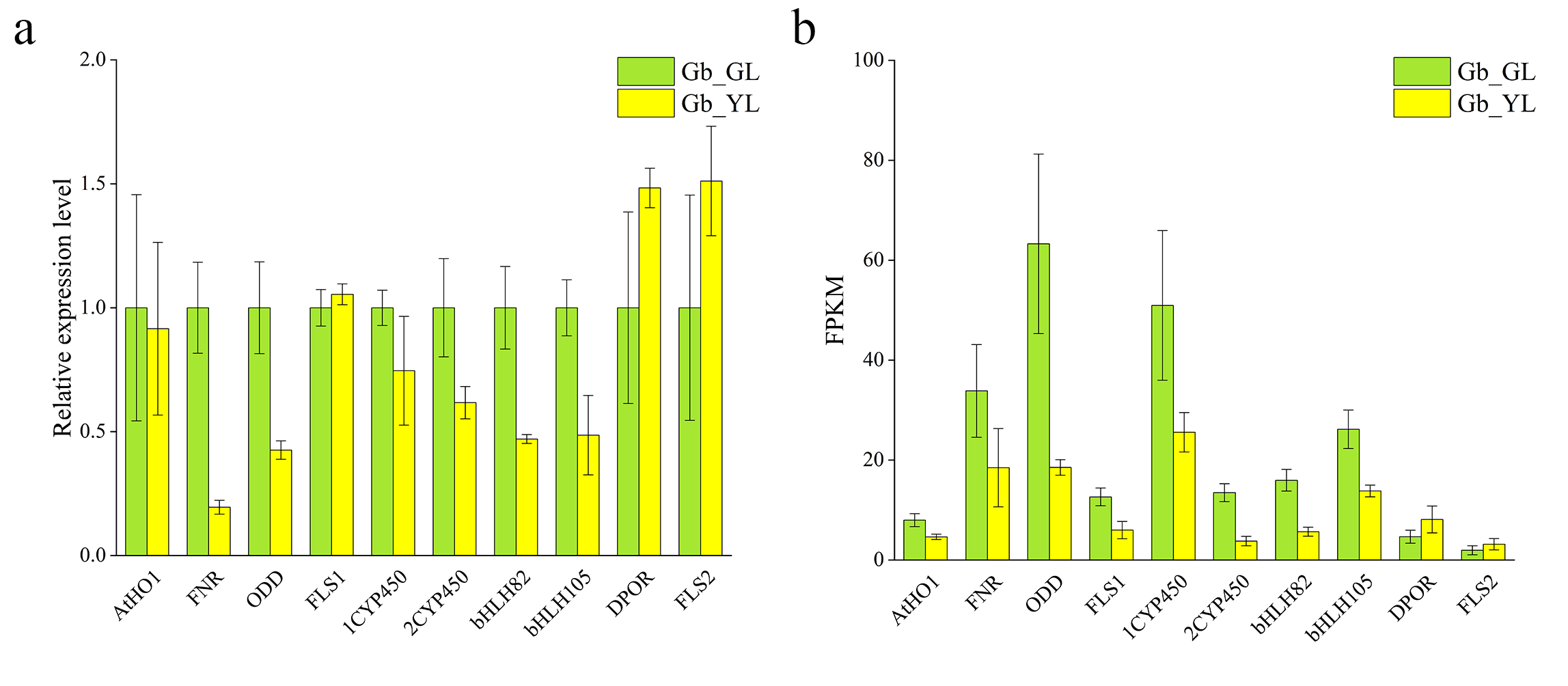

Supplement: Supplementary file 4 — Additional file 4: Figure S3. qRT-PCR validation of 10 putative genes in the ginkgo transcriptome. a. Histogram showing the qPCR results of 10 unigenes between green leaves and yellow leaves of ginkgo. b. Histogram showing the FPKM values of these unigenes. [file 12864_2020_7259_MOESM4_ESM.tif]
